# A genomic mutation signature predicts the clinical outcomes of immunotherapy and characterizes immunophenotypes in gastrointestinal cancer

**DOI:** 10.1038/s41698-021-00172-5

**Published:** 2021-05-04

**Authors:** Xi Jiao, Xin Wei, Shuang Li, Chang Liu, Huan Chen, Jifang Gong, Jian Li, Xiaotian Zhang, Xicheng Wang, Zhi Peng, Changsong Qi, Zhenghang Wang, Yujiao Wang, Yanni Wang, Na Zhuo, Henghui Zhang, Zhihao Lu, Lin Shen

**Affiliations:** 1 Key Laboratory of Carcinogenesis and Translational Research (Ministry of Education), Department of Gastrointestinal Oncology, Peking University Cancer Hospital and Institute, Beijing, China; 2 Life Sciences Institute, Zhejiang University, Hangzhou, China; 3 Genecast Precision Medicine Technology Institute, Beijing, China; 4 Institute of Infectious Diseases, Beijing Ditan Hospital, Capital Medical University, Beijing, China

**Keywords:** Tumour biomarkers, Gastrointestinal cancer, Cancer microenvironment

## Abstract

The association between genetic variations and immunotherapy benefit has been widely recognized, while such evidence in gastrointestinal cancer remains limited. We analyzed the genomic profile of 227 immunotherapeutic gastrointestinal cancer patients treated with immunotherapy, from the Memorial Sloan Kettering (MSK) Cancer Center cohort. A gastrointestinal immune prognostic signature (GIPS) was constructed using LASSO Cox regression. Based on this signature, patients were classified into two subgroups with distinctive prognoses (*p* < 0.001). The prognostic value of the GIPS was consistently validated in the Janjigian and Pender cohort (*N* = 54) and Peking University Cancer Hospital cohort (*N* = 92). Multivariate analysis revealed that the GIPS was an independent prognostic biomarker. Notably, the GIPS-high tumor was indicative of a T-cell-inflamed phenotype and immune activation. The findings demonstrated that GIPS was a powerful predictor of immunotherapeutic survival in gastrointestinal cancer and may serve as a potential biomarker guiding immunotherapy treatment decisions.

## Introduction

Immune checkpoint inhibitors (ICIs) have revolutionized the therapeutic landscape of various cancers, including gastrointestinal cancer. However, only 10–20% of patients respond to ICIs^1,2^, highlighting the urgent need to identify potential biomarkers to screen patients who could benefit from ICIs.

To date, extensive efforts have been made to identify predictive biomarkers of immunotherapy. However, only high microsatellite instability (MSI-H) has been validated in clinical scenarios and programmed death ligand-1 (PD-L1) expression is an important but imperfect predictive biomarker in gastrointestinal cancer with controversial results across different trials^3–9^. Transcriptomic biomarkers such as the T-cell-inflamed gene expression profile (GEP) were shown to be associated with the response to ICIs, but failed to predict survival in gastric or esophageal cancer^10,11^. This may be explained by the application of archival tissue but not fresh tissue, indicating that the limited availability of high-quality mRNA may hinder the clinical utility of transcriptomic biomarkers.

Tumor mutation burden (TMB) is another potential biomarker and a recent study certified the robust association of TMB and response to ICIs^12^. However, TMB remains a controversial biomarker in gastrointestinal cancer^1,13^. Emerging data indicate that not all genetic mutations are equivalent in terms of their immunologic impact. Some mutations, such as ARID1A, TP53, PBRM1, KEAP1, STK11, NOTCH1/2/3, and JAK1/2, may exert positive or negative influences on the outcomes of ICI treatment^14–19^. Nevertheless, all of these mutations are weighted the same in TMB scoring systems, highlighting the limitations of TMB as a predictive biomarker for ICI. Recently, it has been reported that TMB-based survival prediction can be improved by optimizing the TMB algorithm^20^ or by establishing gene mutation-based signatures^21,22^. We therefore investigated the genomic determinants of ICIs benefits in gastrointestinal cancer and developed a gene mutation-based risk model containing the most decisive prognosis-related genes to better predict the clinical outcomes of immunotherapy in patients with gastrointestinal cancer.

## Results

### Clinicopathological features of the patients

In this study, we developed a prediction model based on the Memorial Sloan Kettering (MSK) cohort of 227 gastrointestinal cancer patients who had received ICIs (MSK-GI cohort; esophagogastric cancer, *N* = 118; colorectal cancer, *N* = 109)^13^ and with a median follow-up of 19 months. The prognostic model was validated using data from the Janjigian and Pender cohort^23,24^ and Peking University Cancer Hospital (PUCH) cohorts of 54 and 92 gastrointestinal cancer patients, respectively. Molecular profiling of the tumor samples from the patients was performed by MSK-IMPACT or whole-exome sequencing (WES). Fourteen (25.9%) patients in the Janjigian and Pender cohort and 35 (38%) patients in the PUCH cohort showed durable clinical benefit (DCB), respectively. Supplementary Table 1 provides a summary of the characteristics of the patients in the three cohorts. The flowchart of this study design is shown in Supplementary Fig. 1.

### Construction of the gastrointestinal immune prognostic signature

**Fig. 1:**
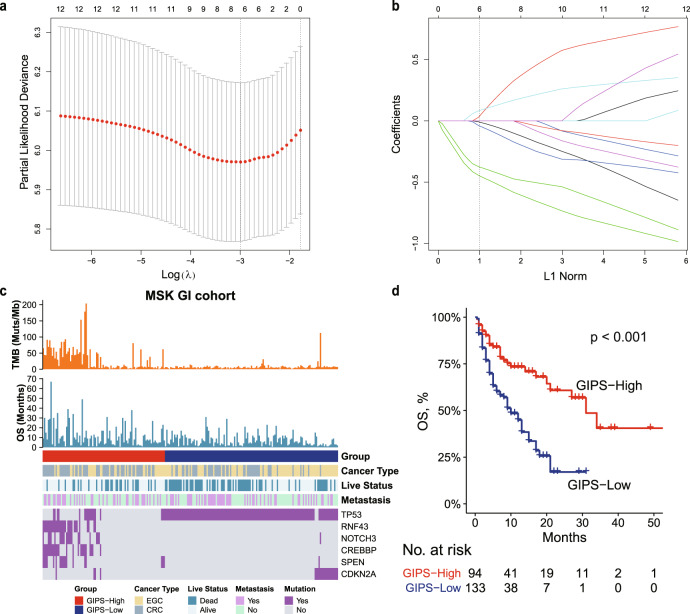
Construction of GIPS. **a** Tenfold cross-validation for selection of tuning parameters in the LASSO regression. Two dotted vertical lines are drawn at the optimal values according to the minimum criterion (right) and the 1 − SE criterion (left). **b** LASSO coefficient profiles of the 12 candidate genes. The dotted vertical line indicates the optimal value (L1 Norm = 1), which was identified by tenfold cross-validation. **c** Heatmap of the clinical and molecular features associated with the GIPS-high and GIPS-low subgroups in the MSK-GI cohort. **d** Kaplan–Meier curves of OS for patients with high and low GIPS scores in the MSK-GI cohort.

The prognostic significance of each gene was first analyzed by the univariate Cox proportional hazards regression model (Supplementary Table 2). To further build a predictive model, genes with *p* < 0.1 and mutation frequency > 8% were selected as seed genes for least absolute shrinkage and selection operator (LASSO) Cox regression with 10-fold cross-validation, which identified 6 genes out of the 12 genes (Fig. 1a, b). A risk model termed gastrointestinal immune prognostic signature (GIPS) was calculated using a formula derived from the mutation status (1 or 0) of the six genes weighted by their regression coefficient. GIPS = (0.558 × *RNF43*) + (0.456 × *CREBBP*) − (0.28 × *CDKN2A*) − (0.154 × *TP53*) + (0.147 × *SPEN*) + (0.082 × *NOTCH3*). In this formula, gene mutation was equivalent to 1 and wild-type status was equivalent to 0. An optimal cutoff value of −0.15 was used to separate the patients into the GIPS-high and GIPS-low groups using X-tile^25^. As expected, the GIPS-high group was associated with better prognosis in the MSK-GI cohort (median overall survival [mOS], 31 months vs. 10 months; *p* < 0.001; hazard ratio (HR), 0.40; 95% confidence interval (95% CI), 0.27–0.59; Fig. 1c, d) and its esophagogastric cancer and colorectal cancer subgroup (Supplementary Fig. 2). Moreover, GIPS served as an independent prognostic factor in the MSK-GI cohort (Table 1).

**Table 1:** Univariate and multivariate Cox analysis for PFS and OS in three cohorts.

Variables	Univariate analysis			Multivariate analysis		
	HR	95% CI	*p*-Value	HR	95% CI	*p*-Value
OS						
MSK-GI cohort^a^						
GIPS (high vs. low)	0.37	0.23–0.58	<0.001	0.33	0.21–0.54	<0.001
TMB (high vs. low)	0.54	0.34–0.86	0.009	0.57	0.35–0.92	0.023
Janjigian and Pender cohort^b^						
GIPS (high vs. low)	0.51	0.27–0.98	0.042	0.07	0.01–0.39	0.002
MSI status (MSI-H vs. MSS)	0.38	0.09–1.60	0.188	0.16	0.03–0.82	0.028
TMB (high vs. low)	0.60	0.29–1.20	0.167	0.45	0.18–1.20	0.099
PD-L1 (positive vs. negative)	0.24	0.08–0.77	0.016	0.09	0.02–0.51	0.006
PUCH cohort^c^						
GIPS (high vs. low)	0.43	0.23–0.80	0.008	0.47	0.25–0.88	0.019
MSI status (MSI-H vs. MSS)	0.47	0.19–1.10	0.097	0.36	0.15–0.88	0.025
TMB (high vs. low)	0.45	0.22–0.93	0.032	0.34	0.16–0.71	0.004
PD-L1 (positive vs. negative)	1.00	0.47–2.20	0.986	1.23	0.55–2.75	0.617
PFS						
Janjigian and Pender cohort^b^						
GIPS (high vs. low)	0.39	0.20–0.74	0.004	0.12	0.03–0.44	0.001
MSI status (MSI-H vs. MSS)	0.39	0.12–1.30	0.123	0.21	0.05–0.85	0.029
TMB (high vs. low)	0.45	0.22–0.91	0.026	0.37	0.15–0.93	0.035
PD-L1 (positive vs. negative)	0.17	0.05–0.64	0.008	0.10	0.02–0.46	0.004
PUCH cohort^c^						
GIPS (high vs. low)	0.42	0.25–0.71	0.001	0.42	0.24–0.72	0.002
MSI status (MSI-H vs. MSS)	0.44	0.22–0.90	0.025	0.33	0.16–0.70	0.004
TMB (high vs. low)	0.47	0.26–0.85	0.013	0.36	0.19–0.67	0.001
PD-L1 (positive vs. negative)	1.30	0.69–2.40	0.415	1.52	0.80–2.90	0.206

^a^The multivariate analysis in the MSK-GI cohort was adjusted for age, sex, cancer type, drug class, and metastasis.

^b^The multivariate analysis in the Janjigian and Pender cohort was adjusted for age, sex, and liver metastasis.

^c^The multivariate analysis in the PUCH cohort was adjusted for age, sex, and drug class.

### Associations of GIPS with clinical benefit, OS, and PFS in the validation cohorts

**Fig. 2:**
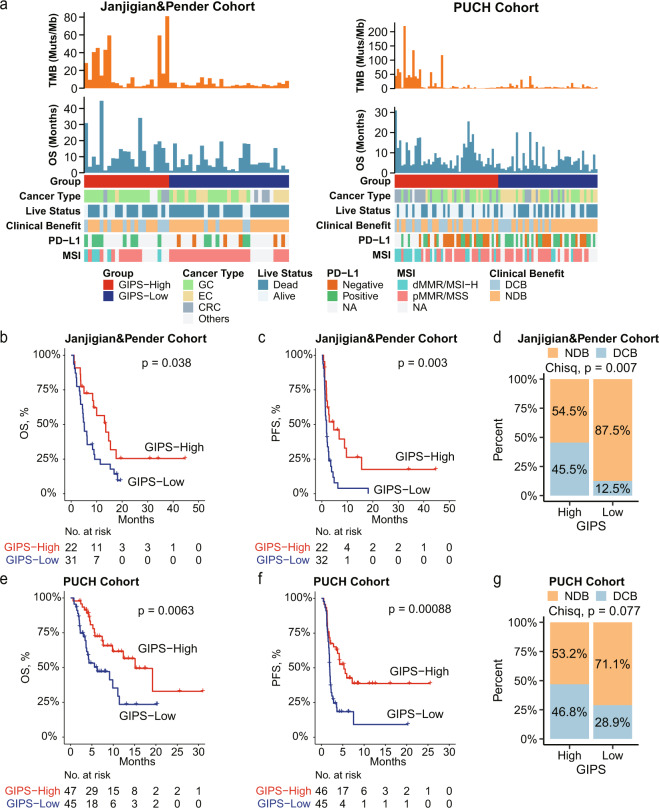
GIPS is a prognostic biomarker and predicts immunotherapeutic benefit in two validation cohorts. **a** Heatmap of the clinical and molecular features associated with the GIPS-high and GIPS-low groups. **b**–**d** Kaplan–Meier curves of OS (**b**) and PFS (**c**), and the rate of durable clinical benefit (**d**) for patients with high and low GIPS scores in the Janjigian and Pender cohort. **e**–**g** Kaplan–Meier curves of OS (**e**) and PFS (**f**), and the rate of durable clinical benefit (**g**) for patients with high and low GIPS scores in the PUCH cohort. For Janjigian and Pender cohort, 53 patients had OS information. For PUCH cohort, 91 patients had PFS information.

To validate the prognostic value of the GIPS model, two independent immunotherapy cohorts of gastrointestinal cancer patients with adequate information on genomic alterations and survival were analyzed. The patients in both cohorts were classified into GIPS-high and GIPS-low groups using the cutoff point obtained from the training cohort. For the Janjigian and Pender cohort of 54 patients with gastrointestinal cancer treated with ICIs, patients with GIPS-high (*N* = 22) had a better overall survival (OS) and progression-free survival (PFS) compared with the GIPS-low patient group (*N* = 32) (mOS, 13.6 months vs. 5.1 months; *p* = 0.038; HR, 0.52; 95% CI, 0.28–0.95; mPFS, 4.7 months vs. 1.9 months; *p* = 0.003; HR, 0.42; 95% CI, 0.23–0.75; Fig. 2a–c). A remarkably higher DCB rate was also displayed in the GIPS-high group (45.5% vs. 12.5%; *p* = 0.007; Fig. 2d). Similarly, in the PUCH cohort, 47 patients were assigned to the GIPS-high group and their OS and PFS were superior to the patients in the GIPS-low group (mOS, 15.1 months vs. 6.2 months; *p* = 0.0063; HR, 0.44; 95% CI, 0.24–0.81; mPFS, 5.3 months vs. 1.9 months; *p* < 0.001; HR, 0.44; 95% CI, 0.26–0.74; Fig. 2a, e, f). GIPS-high patients also demonstrated a higher DCB rate than GIPS-low patients (46.8% vs. 28.9%, *p* = 0.077; Fig. 2g).

### Comparison of GIPS and other potential biomarkers

To explore whether GIPS was a predictive factor for DCB, receiver operating characteristic (ROC) analysis was used to evaluate its predictive value. The ROC analyses of both cohorts demonstrated that GIPS was a predictive biomarker of immunotherapy clinical benefit (Janjigian and Pender cohort: area under the ROC curve [AUC], 0.71; 95% CI, 0.56–0.85; PUCH cohort: AUC, 0.59; 95% CI, 0.49–0.70; Supplementary Fig. 3) and its predictive power was comparable to that of other biomarkers (Supplementary Fig. 3). However, we did not observe significant differences in DCB according to the TMB level (43.8% vs. 18.4%, *p* = 0.11) or MSI status (60% vs. 22.9%, *p* = 0.23) in the Janjigian and Pender cohort or PD-L1 expression in the PUCH cohort (35.7% vs. 36.4%, *p* = 0.96; Supplementary Fig. 4), suggesting that the use of these biomarkers may not be effective.

We next performed univariate and multivariate analyses of these potential biomarkers. The results showed that GIPS remained a powerful and independent prognostic factor for OS and PFS in all three cohorts (all *p*-values < 0.05) (Table 1). However, PD-L1 and MSI-H revealed mixed prognostic value results (Table 1 and Supplementary Fig. 5), indicating a lack of generalizability of these biomarkers, as shown in previous studies^7,8,13,26,27^. Besides, TMB was found to be a robust predictive biomarker across the cohorts (Supplementary Figs. 2 and 5). However, GIPS showed a great power for predicting patient survival, as revealed by the low HR and statistical significance of the survival analysis in each cohort. Overall, GIPS may serve as a powerful predictor of the immunotherapeutic outcomes in gastrointestinal cancer.

### The joint utility of GIPS and TMB for patient stratification and clinical outcome prediction

The relationship between the GIPS and other molecular factors was investigated. Higher proportions of PD-L1 positivity were identified in the GIPS-high subgroup compared with the GIPS-low subgroup in the Janjigian and Pender cohort; however, this correlation was not significant in the PUCH cohort (Supplementary Fig. 6a). Notably, MSI-H/mismatch repair deficient (dMMR) tumors were more frequently GIPS-high in our enrolled datasets (Supplementary Fig. 6b). Interestingly, we also observed a moderate correlation between TMB and GIPS in the MSK-GI (*r* = 0.41, *p* < 0.001) and in the Janjigian and Pender cohorts (*r* = 0.33, *p* = 0.017), and this correlation was lacking in the PUCH cohort (*r* = 0.19, *p* = 0.065, Supplementary Fig. 6c).

**Fig. 3:**
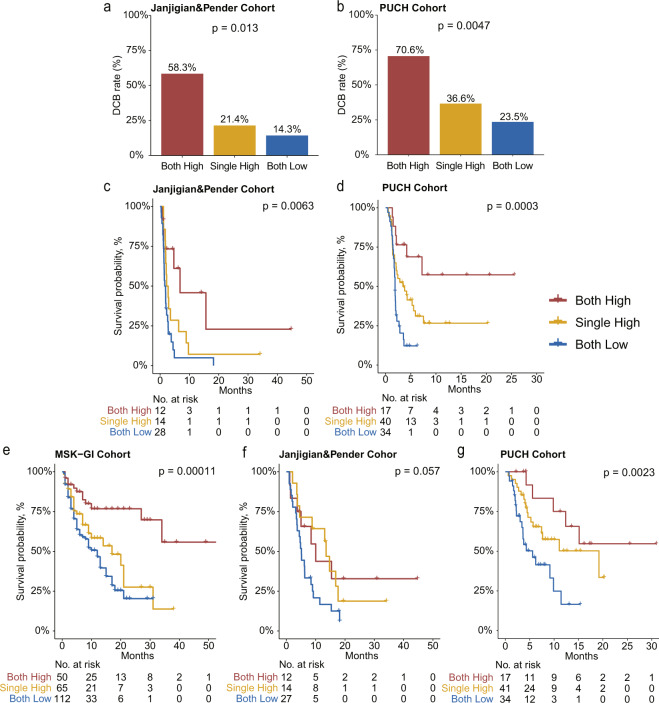
The joint utility of GIPS and TMB in predicting the clinical outcomes of patients with gastrointestinal cancer receiving ICI treatment. **a**, **b** Proportion of patients with DCB calculated within each of the three indicated subgroups. **c**, **d** Kaplan–Meier survival analysis of PFS among patients within each of the three indicated subgroups in the Janjigian and Pender (**c**) and in the PUCH (**d**) cohorts. **e**–**g** Kaplan–Meier survival analysis of OS among patients within each of the three indicated subgroups in the MSK-GI (**e**), Janjigian and Pender (**f**), and PUCH (**g**) cohorts. For Janjigian and Pender cohort, 53 patients had OS information. For PUCH cohort, 91 patients had PFS information.

We further investigated the joint utility of GIPS and TMB for patient stratification and prediction of the clinical outcome. The GIPS-high/TMB-high (both high) subgroup had a remarkably higher DCB rate compared with the subgroup where both were low in the two validation cohorts (Fig. 3a, b). Besides, the median PFS times of the GIPS-high/TMB-high (both high) subgroup were significantly longer compared with the other two subgroups in the Janjigian and Pender cohort and in the PUCH cohort (Fig. 3c, d). The GIPS-high/TMB-high (both high) subgroup also had significantly prolonged OS compared with the other two subgroups in the MSK-GI and PUCH cohorts (Fig. 3e, g). This stratification effect was robustly consistent in the Janjigian and Pender cohort (Fig. 3f). These findings indicated that a combined biomarker based on GIPS and TMB exhibited better predictive value for favorable ICI benefit.

### GIPS-high tumors facilitate favorable immune-cell infiltration and interferon-associated gene signatures

**Fig. 4:**
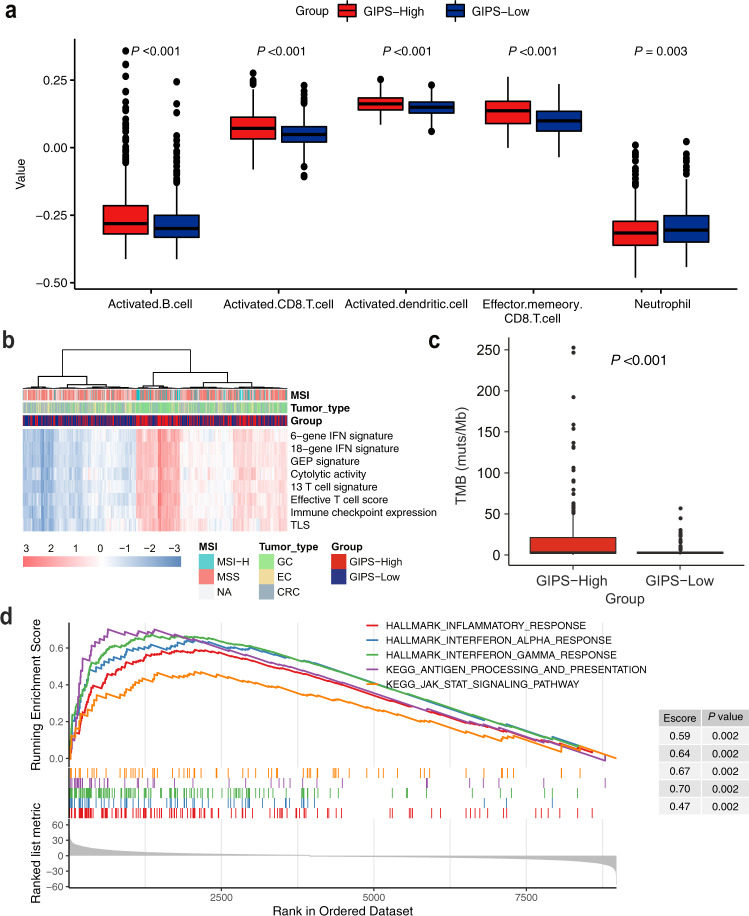
Associations of tumor immune environment features with GIPS in the TCGA cohort. **a** Comparison of immune-cell infiltration according to GIPS status. **b** Heatmap of immune-related signatures in gastrointestinal cancers with GIPS-high and GIPS-low scores. **c** Comparison of nonsynonymous mutations between the GIPS-high and GIPS-low groups. **d** GSEA plot of immune-related pathways in comparisons between the GIPS-high and GIPS-low groups. Abbreviations: IFN, interferon; TLS, tertiary lymphoid structure.

Based on the above results, we hypothesized that GIPS may be an indicator of tumor immune microenvironment features in gastrointestinal cancer patients. The Cancer Genome Atlas (TCGA) gastrointestinal cancer cohort was stratified into GIPS-high and GIPS-low groups based on the GIPS stratification system. Using the single-sample gene set enrichment analysis (ssGSEA) methodology, the degree of infiltrated immune cells was estimated. Compared with GIPS-low tumors, GIPS-high tumors were more infiltrated by immune effector cells, such as effector T cells, dendritic cells, and B cells, but had a low number of neutrophils (Fig. 4a).

Notably, GIPS-high tumors also exhibited a significant enrichment in immune-related signatures (Fig. 4b and Supplementary Fig. 7). Among the enhanced signatures, the 6-gene interferon (IFN) signature, 18-gene IFN signature, and GEP were previously reported to predict the response to ICI therapy^28,29^. GIPS-high tumors displayed significantly more nonsynonymous mutations compared with the GIPS-low tumors (Fig. 4c). GSEA analysis was performed to identify pathways enriched in specific GIPS statuses and the results showed that the pathways of IFN response, antigen processing, presentation mechanism, and inflammatory response were significantly upregulated in GIPS-high tumors (Fig. 4d). These findings indicate that GIPS can predict T-cell inflammation in gastrointestinal cancer and partially explain the correlation between GIPS and immunotherapy benefits.

## Discussion

**Fig. 5:**
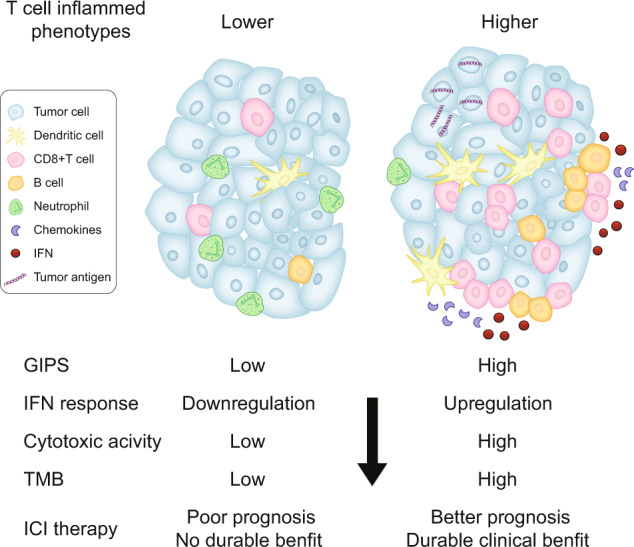
Graphic abstract. The GIPS model plays a role in predicting the tumor immune phenotype and clinical outcomes of ICI treatment in gastrointestinal cancer.

In this study, we developed and validated a genomic classifier, GIPS, consisting of six genes that can better predict the efficacy of ICI therapy in gastrointestinal cancer patients. Our results showed that this signature could stratify patients into benefited and non-benefited subgroups, and served as a strong prognostic factor for gastrointestinal cancer patients treated by ICIs. Besides, GIPS increases cost-effectiveness by offering a smaller panel of genes that can be easily translated into an easy-to-use clinical assay. Furthermore, patients with different GIPS scores had distinct tumor immune microenvironment characteristics. The activation of the antitumor immune response was identified as a potential mechanism underlying the predictive value of GIPS-high in the gastrointestinal cancer population (Fig. 5).

Currently, extensive efforts have been made to identify predictive markers of the response to ICI therapies. However, MSI-H occurs in 0–5% of all metastatic gastrointestinal cancers^6,30^, limiting the use of ICI-based therapy in this population. PD-L1 expression failed to predict response or survival in colorectal cancer^9^, and the survival benefits of ICIs in esophageal and gastric cancer were observed irrespective of PD-L1 expression^4,5,31^. TMB also encounters several issues, including the lack of consensus regarding the cutoff point and the distinct platform of conducting exon sequencing^32^. These factors may account for the mixed results of the prognostic value of TMB reported in different studies^1,8,13^, as displayed in our study. Emerging evidence indicates that some specific genetic mutations exert strong effects on the generation of neoantigens and in shaping the tumor immune microenvironment, ultimately contributing to distinct immune responses^33^. As previously reported, mutations in DNA damage response (DDR) genes and insertion–deletion genetic variants are more likely to generate neoantigens^34^. Moreover, some specific genetic aberrations, such as mutations of TP53, PTEN, NOTCH1/2/3, PBRM1, STK11, and deficits in IFN-γ pathway have been demonstrated to have an impact on immune-cell infiltration and function, and clinical outcomes of ICI therapy^14–16,35–38^. However, current TMB calculations weigh each genetic mutation the same, which is not precise. Recent studies have suggested that the predictive power of TMB might be enhanced by inferring mutational signatures directly from the TMB data^20–22^. In this study, we interrogated the genomic mutations correlated with the prognosis of ICIs in gastrointestinal cancer patients and integrated multiple decisive prognostic genetic parameters into one risk model. As a result, our GIPS model outperformed the existing biomarkers, indicating that GIPS was a good biomarker with more prognostic power.

The AUC for the correlation of GIPS with DCB was moderate. We therefore explored the joint predictive utility of GIPS and TMB, and found that patients with GIPS-high and TMB-high showed the best DCB and the longest survival time in both the validation cohorts. These results suggest that GIPS could identify patients who may not benefit from ICIs, despite having a high TMB, supporting its potential use as a combinatorial biomarker together with TMB for patient stratification.

The immune-modulating function and prognostic value of the six genes in the GIPS classifier for immunotherapy have been elucidated in previous studies^16,19,33,39,40^. However, these genes have not been well characterized in gastrointestinal cancer. *TP53* is frequently mutated in gastrointestinal cancer. Apart from the classic mechanisms of carcinogenesis mediated by *TP53* mutation, the induction of an impaired immune response was observed in *TP53*-mutated gastric cancer, hepatocellular carcinoma, and melanoma^41–43^. Notably, an opposite phenomenon has been observed in lung cancer^44,45^, highlighting that the regulation of tumor immunity by *TP53* is cancer type-dependent. Besides TP53, *CDKN2A* is another senescence-inducing cell cycle regulator required for cancer immune control^40^. The loss of *CDKN2A* function contributes to ICI therapy resistance in experimental and clinical studies^40,46,47^. *NOTCH3* mutation has been recently reported to be related to the activation of the DDR system, which confers sensitivity to immunotherapy in lung cancer^16^. Moreover, *RNF43* and *CREBBP* mutations have been frequently observed in MSI-H gastric cancer or colorectal cancer^48–50^, indicating a favorable immune context in these tumors. As a result, GIPS holds a significant promise in predicting immunotherapy efficacy, as almost all of the genes play critical roles in modulating the immune microenvironment. Further investigation validated that the GIPS-high group can be considered an immune-inflamed phenotype, with immune pathway activation, effector immune-cell infiltration, and higher TMB. These results demonstrate that the GIPS stratification system may provide important insights into the immunologic profile of gastrointestinal cancer.

Our study has some limitations, which are mainly related to its retrospective nature and the inclusion of a heterogeneous group of several gastrointestinal cancer types. Second, the small number of patients with each cancer type in the validation cohorts restricted our ability to analyze individual tumor histology. Third, patients in the PUCH cohort were treated using different anti-programmed cell death-1 (PD-1)/PD-L1 antibodies from various pharmaceutical companies, which might result in drug bias. A prospective study with a larger sample size of gastrointestinal cancer patients treated by one specific ICI is warranted to assess the predictive value of GIPS in the future.

In summary, our six-gene GIPS model is a promising prognostic and predictive biomarker of the therapeutic benefit of ICIs in gastrointestinal cancer. Furthermore, this signature offers a cost-effective approach to facilitate the identification of potential responders to immunotherapy that can hopefully be further validated in a prospective study.

## Methods

### Study design and population

This multicohort study consisted of a three-step approach (biomarker discovery, biomarker validation, and mechanism exploration). The study design is shown in Supplementary Fig. 1. We obtained genomic and clinical data from four cohorts of gastrointestinal cancer patients treated with ICIs from publicly available datasets of the MSK Cancer Center (http://www.cbioportal.org/)^13,23^, British Columbia Cancer Agency^24^, and from our real-world dataset of PUCH. The MSK cohort containing 236 patients with gastrointestinal cancer was referred as the training cohort (MSK-GI) and was used to screen for genetic parameters with potential prognostic value to construct a prognostic model^13^. Nine tumor samples were excluded because of the unavailability of their genetic variants. In the subsequent clinical validation phase, we employed three immunotherapeutic cohorts as follows: (1) the Janjigian cohort, containing 40 metastatic, chemotherapy-refractory esophagogastric cancer patients treated with a PD-1 inhibitor alone or in combination with cytotoxic T-lymphocyte-associated protein-4 (CTLA-4) inhibitor^23^; (2) the Pender cohort, with 14 patients with metastatic or advanced gastrointestinal cancer, who were treated with anti-PD-1/PD-L1 antibodies alone or in combination with anti-CTLA-4 antibodies between April 2014 and August 2018^24^; and (3) the PUCH cohort, including 92 patients with gastrointestinal cancer and treated with anti-PD-1/PD-L1 antibodies alone or in combination with anti-CTLA-4 antibodies between August 2015 and May 2019. The details of the patient selection criteria are presented in the Supplementary Methods. In addition, the TCGA cohort of gastrointestinal cancer (esophageal cancer, *N* = 184; gastric cancer, *N* = 439; colorectal cancer, *N* = 380) was used to explore whether our model could capture the features of the tumor immune microenvironment. The patient characteristics of the three immunotherapeutic cohorts are shown in Supplementary Table 1.

This study was approved by the Institutional Review Board at the PUCH (2020MS01) and was conducted under the Declaration of Helsinki. For the three publicly available cohorts, institutional review board approvals at MSK Cancer Center and the University of British Columbia BC Cancer Research were also obtained^13,23,24^.

### Outcome

Tumor responses were determined by a clinical radiographic assessment based on the Response Evaluation Criteria in Solid Tumors 1.1. DCB was defined as complete response, partial response, or stable disease (SD) lasting ≥24 weeks; no durable benefit was defined as progressive disease or SD lasting <24 weeks^51^. In the immunotherapeutic cohorts, OS or PFS was used as the survival endpoints.

### Next-generation sequencing

Tumor tissues from the MSK-GI cohort and the Janjigian and Pender cohort were profiled with MSK-IMPACT sequencing (341-gene panel, 410-gene panel, or 468-gene panel) or whole-genome sequencing. Tissue processing and sequencing data analysis were performed as previously described^13,23,24^. Germline variants were also identified through the concurrent sequencing of patient-matched DNA from peripheral blood samples. The mutation data of each sample were obtained from the cBioPortal and previously published articles^13,23,24^.

In the PUCH cohort, WES was performed with Illumina NovaSeq on tumor formalin-fixed paraffin-embedded (FFPE) samples and matched white blood cell samples of the patients. Detailed information on the WES process is presented in the Supplementary Methods. TMB was determined by analyzing nonsynonymous somatic mutations per megabase. The cutoff value for stratifying TMB-high and TMB-low of the immunotherapeutic cohorts was defined as the top 30% of the TMB in each cohort.

### MSI/mismatch repair status testing

In the PUCH cohort, the MMR status was assessed by immunohistochemical (IHC) staining using monoclonal antibodies for anti-mutL homolog 1 (Clone ES05), anti-mutS homolog 2 (Clone 25D12), anti-mutS homolog 6 (Clone EP49), and anti-postmeiotic segregation increased 2 (Clone EP51). Tumors lacking the expression of any one of the four proteins were considered dMMR; otherwise, they were considered MMR proficient. The MSI status was measured by PCR-based molecular testing, including five microsatellite loci as follows: BAT-25, BAT-26, D2S123, D5S346, and D17S250. MSI-H tumors were defined as instability at two or more of these markers. In the Janjigian and Pender cohort, MSI status was assessed using the MSIsensor algorithm, with MSI-H defined as an MSIsensor score ≥ 10^23^.

### IHC staining for PD-L1

PD-L1 expression in the PUCH cohort was assessed by IHC staining of FFPE sections using an anti-PD-L1 antibody (rabbit, clone SP142, 1 : 100; Spring Bioscience, CA, USA). The PD-L1 expression of tumor samples was centrally assessed by two pathologists. PD-L1 positivity was defined as the presence of staining cell percentage ≥ 1% of tumor cells and immune cells.

### Construction of the GIPS

Univariate Cox regression analyses assessed the association between each gene mutation (mutation frequency > 8%) and survival, and genes with *p* < 0.1 were selected as candidate genes. To further narrow the scope of the candidate genes and to prevent overfitting, the LASSO Cox regression algorithm with the glmnet package (v3.0-2) was adopted to build an optimal model with the minimum number of genes^52^. The penalty parameter was estimated by tenfold cross-validation with the minimum partial likelihood deviance. We utilized X-tile software to generate the appropriate cutoff values to stratify patients into GIPS-high and GIPS-low groups^25^.

### mRNA expression profiling analysis

The associations between GIPS and immune-related features were analyzed based on the TCGA datasets, which had both DNA-sequencing and RNA-sequencing (RNA-seq) data available on the website (https://gdc.cancer.gov/about-data/publications/pancanatlas). The expression data for mRNA in RNA-Seq by Expectation-Maximization (RSEM) values were transformed to log_10_(RSEM + 1). We used previously published immune-related signatures to characterize the tumor immune microenvironment (Supplementary Table 3). The signature scores of each patient were calculated by averaging the included genes in the corresponding signature gene sets. To quantify the proportions of immune cells in the tumor microenvironment, we implemented the ssGSEA using the GSVA package, which allows the prediction of the distributions of multiple immune cell types in tumor tissues^53,54^. GSEA was performed on the differentially expressed genes between the GIPS-high and GIPS-low groups, which were screened using the edgeR package. A ranked list of genes from the edgeR output was created using −log_10_(*p*-value) × sign(log(fold change)). The R package clusterProfiler was applied to the ranked gene list to perform GSEA based on the Molecular Signatures Database C2 and Hallmark gene set^55,56^.

### Statistical analysis

The data were analyzed using R statistical software version 3.6.1 and SPSS software version 23.0. Categorical data were compared using the *χ*^2^-test or Fisher’s exact test, as appropriate. Kaplan–Meier survival curves were assessed using Log-rank test for the OS and PFS estimations. ROC analysis was used to assess the predictive accuracy of GIPS and other predictors. Group means were compared by Student’s *t*-test for normally distributed data and nonparametric tests were used when the data were not normally distributed. *p* < 0.05 was considered as statistically significant.

### Reporting summary

Further information on research design is available in the Nature Research Reporting Summary linked to this article.

## Supplementary information

Reporting Summary

Supplementary Information

## Data Availability

The data generated and analyzed during this study are described in the following data record: 10.6084/m9.figshare.14303075^57^. The datasets of the MSK-GI, Janjigian and Pender, PUCH, and TCGA cohort generated and analyzed during the current study have been deposited at 10.6084/m9.figshare.14174807.v2^58^ and 10.6084/m9.figshare.14174828^59^ in the files “MSK-GI_JP_PUCH_clinical_info_with_GIPS.xlsx” and “TCGA_clinical_info_with_GIPS_and_immune_signatures(2).xlsx,” respectively. The genomic and clinical data of the MSK-GI cohort, Janjigian and Pender cohort, and PUCH cohort are openly available and were downloaded from the following places: http://www.cbioportal.org/study?id=tmb_mskcc_2018, https://www.cbioportal.org/study/summary?id=egc_msk_2017, http://clincancerres.aacrjournals.org/content/27/1/202.article-info, https://www.bcgsc.ca/downloads/immunoPOG/, and 10.6084/m9.figshare.14168879^60^. The DNA-seq and RNA-seq data of the TCGA data used in Fig. 4 and Supplementary Fig. 7 can be downloaded from https://gdc.cancer.gov/about-data/publications/pancanatlas in files “EBPlusPlusAdjustPANCAN_IlluminaHiSeq_RNASeqV2.geneExp.tsv” and “mc3.v0.2.8.PUBLIC.maf.gz.”
