# Supplementary material for: A genomic mutation signature predicts the clinical outcomes of immunotherapy and characterizes immunophenotypes in gastrointestinal cancer
Source: NPJ Precis Oncol. 2021 May 4;5:36. doi: 10.1038/s41698-021-00172-5 (PMC8096820; doi:10.1038/s41698-021-00172-5)
Supplement: Supplementary file 1 — Reporting Summary [file 41698_2021_172_MOESM1_ESM.pdf]

nature research

Corresponding author(s): Lin Shen; Zhihao Lu

Last updated by author(s): Xi Jiao

Reporting Summary

Nature Research wishes to improve the reproducibility of the work that we publish. This form provides structure for consistency and transparency in reporting. For further information on Nature Research policies, see our Editorial Policies and the Editorial Policy Checklist.

Statistics

For all statistical analyses, confirm that the following items are present in the figure legend, table legend, main text, or Methods section.

n/a	Confirmed	
☐	☒	The exact sample size (*n*) for each experimental group/condition, given as a discrete number and unit of measurement
☐	☒	A statement on whether measurements were taken from distinct samples or whether the same sample was measured repeatedly
☐	☒	The statistical test(s) used AND whether they are one- or two-sided<br>*Only common tests should be described solely by name; describe more complex techniques in the Methods section.*
☐	☒	A description of all covariates tested
☐	☒	A description of any assumptions or corrections, such as tests of normality and adjustment for multiple comparisons
☐	☒	A full description of the statistical parameters including central tendency (e.g. means) or other basic estimates (e.g. regression coefficient) AND variation (e.g. standard deviation) or associated estimates of uncertainty (e.g. confidence intervals)
☐	☒	For null hypothesis testing, the test statistic (e.g. *F*, *t*, *r*) with confidence intervals, effect sizes, degrees of freedom and *P* value noted<br>*Give P values as exact values whenever suitable.*
☒	☐	For Bayesian analysis, information on the choice of priors and Markov chain Monte Carlo settings
☐	☒	For hierarchical and complex designs, identification of the appropriate level for tests and full reporting of outcomes
☒	☐	Estimates of effect sizes (e.g. Cohen's *d*, Pearson's *r*), indicating how they were calculated

Our web collection on statistics for biologists contains articles on many of the points above.

Software and code

Policy information about availability of computer code

Data collection

We obtained genomic and clinical data from three cohorts of gastrointestinal cancer patients treated with ICIs. Three were publicly available datasets of the Memorial Sloan Kettering (MSK) Cancer Center (http://www.cbioportal.org/study?id=tmb_mskcc_2018; https://www.cbioportal.org/study/summary?id=egc_msk_2017; http://clincancerres.aacrjournals.org/content/27/1/202.article-info). One was our own real-world dataset of Peking University Cancer Hospital (PUCH).

Data analysis

R version 3.6.1 (2019-07-05)
Platform: x86_64-w64-mingw32/x64 (64-bit)
Running under: Windows 10 x64 (build 18362)

R packages:
RColorBrewer_1.1-2 mclust_5.4.5 Biobase_2.46.0 BiocGenerics_0.32.0
GSVA_1.34.0 genefilter_1.68.0 foreach_1.4.7 data.table_1.12.8
reshape2_1.4.3 dplyr_0.8.3 pROC_1.15.3 ggthemes_4.2.0
survminer_0.4.6 ggpubr_0.2.4 Hmisc_4.3-0 Formula_1.2-3
survival_3.1-8 lattice_0.20-38 ggpmisc_0.3.3 xlsx_0.6.1
readxl_1.3.1 stringr_1.4.0 ggplot2_3.2.1 pheatmap_1.0.12
magrittr_1.5 wo

For manuscripts utilizing custom algorithms or software that are central to the research but not yet described in published literature, software must be made available to editors and reviewers. We strongly encourage code deposition in a community repository (e.g. GitHub). See the Nature Research guidelines for submitting code & software for further information.

Data

Policy information about availability of data

All manuscripts must include a data availability statement. This statement should provide the following information, where applicable:

- Accession codes, unique identifiers, or web links for publicly available datasets
- A list of figures that have associated raw data
- A description of any restrictions on data availability

The genomic and clinical data of the MSK-GI cohort, Janjigian & Pender and PUCH cohorts were available at the websites (http://www.cbioportal.org/study?id=tmb_mskcc_2018; https://www.cbioportal.org/study/summary?id=egc_msk_2017; http://clincancerres.aacrjournals.org/content/27/1/202.article-info; https://www.bcgsc.ca/downloads/immunoPOG/; https://doi.org/10.6084/m9.figshare.14168879). The datasets of the MSK-GI, Janjigian & Pender, PUCH and TCGA cohort generated and/or analyzed during the current study have been deposited at https://doi.org/10.6084/m9.figshare.14174807.v2 and https://doi.org/10.6084/m9.figshare.14174828.

Field-specific reporting

Please select the one below that is the best fit for your research. If you are not sure, read the appropriate sections before making your selection.

☒ Life sciences ☐ Behavioural & social sciences ☐ Ecological, evolutionary & environmental sciences

For a reference copy of the document with all sections, see nature.com/documents/nr-reporting-summary-flat.pdf

Life sciences study design

All studies must disclose on these points even when the disclosure is negative.

Sample size	Sample size was determined based on the the number of patients available retrospectively that met the inclusion criteria.<br>(1) The MSK cohort containing 236 patients with gastrointestinal cancer was referred as the training cohort (MSK-GI) to screen genetic parameters with potential prognostic value and to construct the prognostic model.<br>(2) The Janjigian cohort, containing 40 metastatic, chemotherapy-refractory esophagogastric cancer patients treated with a programmed cell death-1 (PD-1) inhibitor alone or in combination with cytotoxic T-lymphocyte-associated protein-4 (CTLA-4) inhibitor.<br>(3) The Pender cohort, with 14 patients with metastatic or advanced gastrointestinal cancer who were treated with anti-PD-1/PD-L1 antibodies alone or in combination with anti-CTLA-4 antibodies between April 2014 and August 2018.<br>(4) The PUCH cohort, including 92 patients with gastrointestinal cancer, and treated with anti-PD-1/PD-L1 antibodies alone or in combination with anti-CTLA-4 antibodies between August 2015 and May 2019.<br>(5) The Cancer Genome Atlas (TCGA) cohort of gastrointestinal cancer (esophageal cancer, N = 184; gastric cancer, N = 439; colorectal cancer, N = 380) was used to explore whether our model could capture the features of the tumor immune microenvironment.
Data exclusions	Nine tumor samples were excluded in MSK cohort because of the unavailability of their genetic variants.
Replication	We have validated our results by reanalyzing involved data with several different analytic approaches .
Randomization	No randomization was performed as this was a retrospective study.
Blinding	Blinding was not possible for the analysis as it was done retrospectively and there was no subjective analysis that was performed that could be biased by knowledge.

Reporting for specific materials, systems and methods

We require information from authors about some types of materials, experimental systems and methods used in many studies. Here, indicate whether each material, system or method listed is relevant to your study. If you are not sure if a list item applies to your research, read the appropriate section before selecting a response.

Materials & experimental systems

n/a	Involved in the study	
☒	☐	Antibodies
☒	☐	Eukaryotic cell lines
☒	☐	Palaeontology and archaeology
☒	☐	Animals and other organisms
☐	☒	Human research participants
☐	☒	Clinical data
☒	☐	Dual use research of concern

Methods

n/a	Involved in the study	
☒	☐	ChIP-seq
☒	☐	Flow cytometry
☒	☐	MRI-based neuroimaging

Human research participants

Policy information about studies involving human research participants

Population characteristics	See above.
Recruitment	This study was done retrospectively and no patients were directly recruited prospectively.
Ethics oversight	This study was approved by the Institutional Review Board at the Peking University Cancer Hospital (2020MS01) and conducted under the Declaration of Helsinki. For the three publicly available cohorts, institutional review board approvals at Memorial Sloan Kettering Cancer Center and the University of British Columbia BC Cancer Research were also obtained.

Note that full information on the approval of the study protocol must also be provided in the manuscript.

Clinical data

Policy information about clinical studies

All manuscripts should comply with the ICMJE guidelines for publication of clinical research and a completed CONSORT checklist must be included with all submissions.

Clinical trial registration	*Provide the trial registration number from ClinicalTrials.gov or an equivalent agency.*
Study protocol	*Note where the full trial protocol can be accessed OR if not available, explain why.*
Data collection	*Describe the settings and locales of data collection, noting the time periods of recruitment and data collection.*
Outcomes	*Describe how you pre-defined primary and secondary outcome measures and how you assessed these measures.*
