# Supplementary material for: A genomic mutation signature predicts the clinical outcomes of immunotherapy and characterizes immunophenotypes in gastrointestinal cancer
Source: NPJ Precis Oncol. 2021 May 4;5:36. doi: 10.1038/s41698-021-00172-5 (PMC8096820; doi:10.1038/s41698-021-00172-5)
Supplement: Supplementary file 2 — Supplementary Information [file 41698_2021_172_MOESM2_ESM.pdf]

Supplementary materials

A novel genomic mutation signature predicts clinical outcomes of immunotherapy and characterizes immunophenotypes in gastrointestinal cancer

Contents:

Methods

Supplementary table 1-3

Supplementary figure 1-7

Supplementary Methods

Study design and population

We identified 92 patients in our PUCH cohort, who met the following following criteria: 1) metastatic GI cancer patients who had failed the standard therapy; 2) patients who received at least one cycle of a PD-1/PD-L1 inhibitor, alone or combined with other agents between August 2015 and May 2019, within the context of immunotherapeutic clinical trial (NCT02825940, NCT02978482, NCT03195478, NCT02915432, NCT03167853, NCT03472365, NCT02872116, CTR20160872, NCT03713905, NCT03736889 and NCT03667170); 3) patients with complete medical information; and 4) patients with eligible tissue and blood samples for WES sequencing.

Next-generation sequencing

In PUCH cohort, we extracted DNA from FFPE tissue and matched blood cell using the blackPREP FFPE DNA Kit (Analytik Jena AG, Jena, Germany) and the Tiangen Whole Blood DNA Kit (Tiangen, Beijing, PRC). Extracted DNA was subsequently sheared into fragments by a Covaris M220 Focused-Ultrasonicator (Covaris, Massachusetts, USA) with a peak at 200 bp. The KAPA HTP Library Preparation Kit was prepared for the DNA library construction. DNA libraries were captured using a NimbleGen 44M human exome array, and then were subjected to Illumina NovaSeq for paired-end sequencing.

Sequencing data were mapped to the reference genome (hg19) using the BWA programs [1]. Variants were called using VarDict and FreeBayes [2]. The functional annotation of the genetic variants was performed with the ANNOVAR assay [3]. Somatic variant was identified with the following criteria: (1) the mutations of FFPE tumor samples were blanked by paired blood cell samples from patients; (2) located in intergenic regions or intronic regions; (3) synonymous SNVs; (4) depth < 40; (5) allele frequency < 0.03; and (6) allele frequency ≥ 0.002 in the Exome Aggregation Consortium (ExAC) database.

References

1 Li, H. J. a. p. a. Aligning sequence reads, clone sequences and assembly contigs with BWA-MEM. arXiv **1303**, 3997. (2013).

2 Lai, Z. et al. VarDict: a novel and versatile variant caller for next-generation sequencing in cancer research. Nucleic Acids Res **44**, e108 (2016).

3 Wang, K., Li, M. & Hakonarson, H. ANNOVAR: functional annotation of genetic variants from high-throughput sequencing data. Nucleic Acids Res **38**, e164 (2010).

Supplementary table 1. Clinical characteristics of patients from three immunotherapeutic cohorts

Characteristics	MSK-GI cohort (*N*=227)	Janjigian & Pender cohort (*N*=54)	PUCH cohort (*N*=92)
**Age**			
≥65	74 (32.6%)	22 (40.7%)	25 (27.2%)
<65	153 (67.4%)	32 (59.3%)	67 (72.8%)
**Sex**			
Male	152 (67.0%)	39 (72.2%)	67 (72.8%)
Female	75 (33.0%)	15 (27.8%)	25 (27.2%)
**Tumor type**			
Esophageal cancer	50 (22.0%)	13 (24.1%)	27 (29.3%)
Gastric cancer	68 (30.0%)	30 (55.6%)	39 (42.4%)
Colorectal cancer	109 (48.0%)	6 (11.1%)	25 (27.2%)
Others	0	5 (9.3%) [a]	1 (1.1%) [b]
**PD-L1**			
Positive		13 (24.1%)	33 (35.9%)
Negative	NA	6 (11.1%)	28 (30.4%)
NA		35 (64.8%)	31 (33.7%)
**MSI status**			
MSI-H/dMMR		5 (9.3%) [c]	22 (23.9%)
MSS/pMMR	NA	35 (64.8%)	48 (52.2%)
NA		14 (25.9%)	22 (23.9%)
**Drug type**			
PD-1/PD-L1	185 (81.50%)	36 (66.7%)	81 (88.0%) [d]
CTLA-4	3 (1.3%)	0 (0%)	0 (0%)
Combo	39 (17.2%)	18 (33.3%)	11 (12.0%)

Percentages might not total 100% because of rounding. NA, Not available.

a, two pancreatic cancer patients, and three cholangiocarcinoma patients.

b, one cholangiocarcinoma patients.

c, MSIsensor method was used to evaluated MSI status in Janjigian & Pender cohort. Samples with score >= 10 were classified as MSI-H.

d, three patients received PD-1 inhibitor plus chemotherapy and one patient received PD-1 inhibitor plus apatinib.

Supplementary table 2. Univariate Cox analyses of each gene mutation (mutation rate >5%)

gene_symbol	count_mut_ samples	freq_mut_ samples	HR_OS	lower.95_OS	upper.95_OS	Cox_*P*val_OS
RNF43	30	0.13	0.23	0.09	0.57	0.001
CREBBP	25	0.11	0.24	0.10	0.60	0.002
TP53	148	0.65	1.84	1.18	2.88	0.008
NOTCH3	21	0.09	0.23	0.07	0.71	0.01
SPEN	19	0.08	0.29	0.11	0.80	0.02
IRS1	14	0.06	0.09	0.01	0.65	0.02
KMT2C	26	0.11	0.40	0.18	0.86	0.02
PTCH1	22	0.10	0.36	0.14	0.88	0.03
NOTCH4	14	0.06	0.21	0.05	0.84	0.03
ARID1A	47	0.21	0.56	0.32	0.96	0.03
BCOR	17	0.07	0.29	0.09	0.91	0.03
CASP8	15	0.07	0.30	0.09	0.94	0.04
DNMT1	13	0.06	0.23	0.06	0.93	0.04
MTOR	15	0.07	0.30	0.10	0.97	0.05
CDKN2A	22	0.10	1.88	1.00	3.54	0.05
CTCF	12	0.05	0.14	0.02	1.02	0.05
PIK3R1	14	0.06	0.37	0.13	1.01	0.05
FAT1	26	0.11	0.45	0.20	1.03	0.06
ZFHX3	26	0.11	0.50	0.24	1.03	0.06
NF1	24	0.11	0.45	0.20	1.04	0.06
EPHB1	12	0.05	0.27	0.07	1.09	0.07
BRCA1	12	0.05	0.28	0.07	1.13	0.07
BRAF	14	0.06	0.35	0.11	1.11	0.08
CIC	17	0.07	0.45	0.18	1.10	0.08
ANKRD11	15	0.07	0.37	0.12	1.15	0.09
JAK1	12	0.05	0.36	0.11	1.16	0.09
ATRX	18	0.08	0.47	0.19	1.15	0.10
PREX2	12	0.05	2.02	0.88	4.64	0.10
MGA	16	0.07	0.43	0.16	1.18	0.10
B2M	15	0.07	0.44	0.16	1.21	0.11
KRAS	64	0.28	0.71	0.45	1.10	0.13
KMT2B	14	0.06	0.34	0.08	1.37	0.13
ALK	16	0.07	0.46	0.17	1.26	0.13
PIK3CG	15	0.07	0.46	0.17	1.27	0.13
EP300	15	0.07	0.50	0.20	1.26	0.14
PTPRS	29	0.13	0.62	0.33	1.17	0.14
PTPRD	15	0.07	0.43	0.14	1.35	0.15
TET2	17	0.07	0.52	0.21	1.28	0.15
DICER1	16	0.07	0.52	0.21	1.29	0.16
MST1R	12	0.05	0.48	0.18	1.32	0.16
AR	12	0.05	0.44	0.14	1.38	0.16

SETD2	17	0.07	0.52	0.21	1.29	0.16
CTNNB1	18	0.08	0.52	0.21	1.29	0.16
TGFBR2	14	0.06	0.49	0.18	1.34	0.16
LATS2	13	0.06	0.49	0.18	1.34	0.17
POLE	19	0.08	0.58	0.27	1.25	0.17
PTEN	18	0.08	0.53	0.22	1.31	0.17
NOTCH2	12	0.05	0.46	0.15	1.45	0.19
SMARCA4	26	0.11	0.63	0.31	1.26	0.19
TCF7L2	21	0.09	0.60	0.28	1.31	0.20
FBXW7	25	0.11	0.61	0.28	1.31	0.20
DOT1L	16	0.07	0.59	0.26	1.35	0.21
KDM5C	15	0.07	0.53	0.20	1.46	0.22
GRIN2A	12	0.05	0.54	0.20	1.47	0.23
SOX9	19	0.08	0.59	0.24	1.45	0.25
AXIN2	14	0.06	0.56	0.20	1.51	0.25
CARD11	13	0.06	1.52	0.74	3.15	0.25
BRCA2	24	0.11	0.69	0.36	1.33	0.26
ARID1B	26	0.11	0.68	0.34	1.35	0.27
PIK3CA	43	0.19	0.74	0.43	1.29	0.29
IGF1R	17	0.07	1.41	0.71	2.80	0.33
POLD1	15	0.07	0.65	0.26	1.60	0.35
PTPRT	21	0.09	0.72	0.34	1.57	0.41
SMAD4	28	0.12	0.76	0.40	1.46	0.41
RBM10	14	0.06	0.67	0.24	1.82	0.43
KMT2D	45	0.20	0.82	0.49	1.35	0.44
FLT1	14	0.06	0.72	0.29	1.77	0.47
PGR	14	0.06	0.75	0.32	1.71	0.49
MSH2	12	0.05	0.71	0.26	1.93	0.50
ERBB3	19	0.08	0.75	0.33	1.72	0.50
FLT4	16	0.07	0.78	0.36	1.71	0.54
BRD4	12	0.05	1.29	0.56	2.94	0.55
KMT2A	19	0.08	0.81	0.37	1.74	0.58
MSH6	13	0.06	0.79	0.29	2.14	0.64
ATM	17	0.07	1.18	0.57	2.45	0.65
GLI1	19	0.08	0.84	0.39	1.81	0.65
ERBB4	21	0.09	0.86	0.43	1.71	0.67
NCOR1	17	0.07	1.16	0.58	2.30	0.68
PDGFRA	12	0.05	0.84	0.34	2.06	0.70
HLA.A	12	0.05	0.82	0.30	2.24	0.70
NOTCH1	22	0.10	0.89	0.46	1.73	0.73
TSC2	18	0.08	0.88	0.41	1.89	0.74
ARAF	12	0.05	0.87	0.38	2.00	0.74
EPHA5	20	0.09	0.89	0.43	1.83	0.74
ERBB2	17	0.07	0.89	0.39	2.02	0.77

APC	86	0.38	0.96	0.64	1.44	0.85
PBRM1	13	0.06	0.96	0.42	2.19	0.92
TERT	15	0.07	1.04	0.48	2.24	0.92
SOX17	12	0.05	1.02	0.42	2.52	0.96

Supplementary table 3. Public-available gene sigatures used in the study

Signature Name	References
6-gene IFN signature	Ayers M et al, J Clin Invest 2017;127:2930-2940
18-gene IFN signature	Ayers M et al, J Clin Invest 2017;127:2930-2940
Gene Expression Profile	Cristescu R, et al, Science 2018;362
Cytolytic activity	Rooney et al. Cell. 2015;160:48-61
13 T-cell signature	Spranger et al. Proc Natl Acad Sci U S A. 2016;113(48):E7759-E7768.
Effective T cell score	McDermott DF, Nat Med, 2018 ;24(6):749-757
Immune checkpoint expression	Sanchez A, Lancet Oncol. 2020;21(2):283-293
TLS	Finkin et al. Nat Immunol. 2015;16:1235-44

Abbreviations: IFN, interferon; TLS, tertiary lymphoid structure

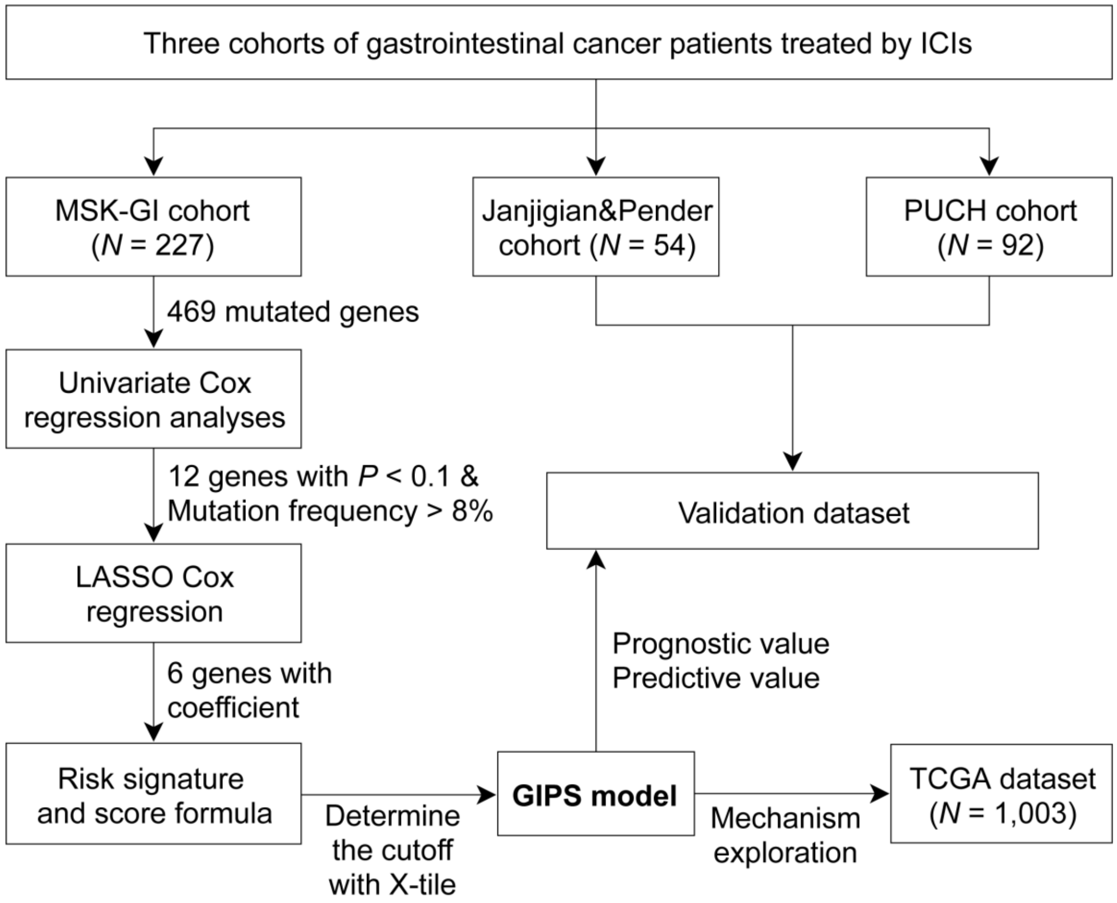

Supplementary Figure 1. The flow chart showing the scheme of our study.

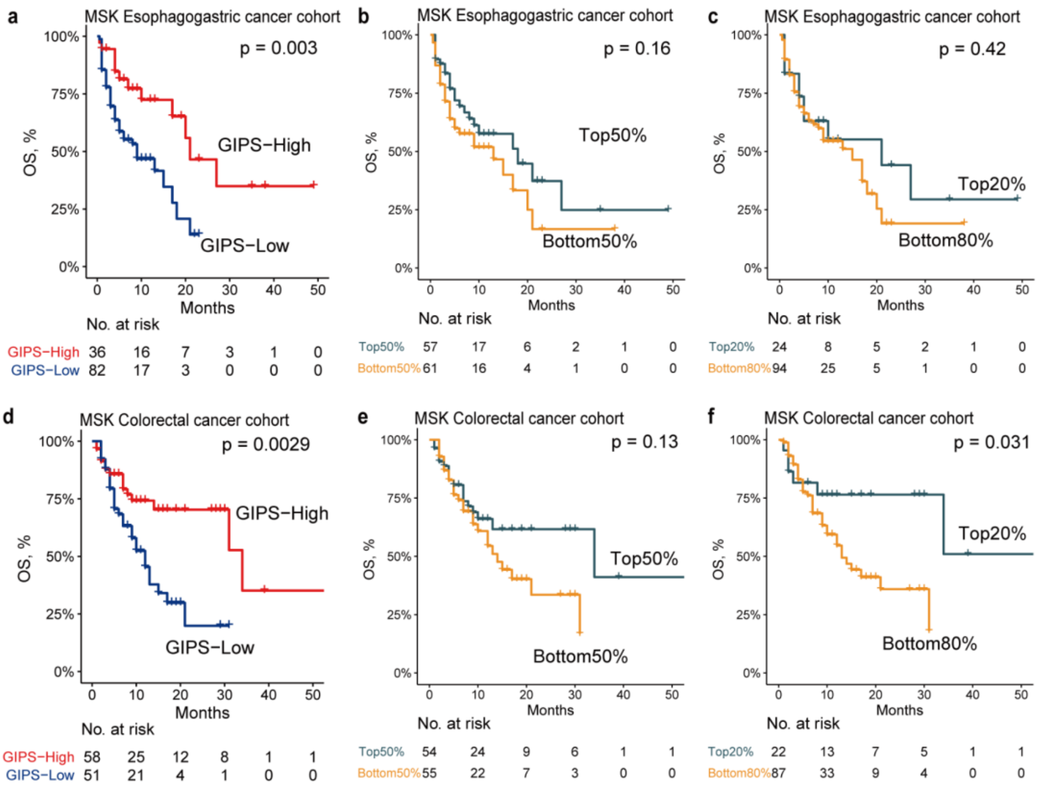

Supplementary Figure 2. Prognostic role of GIPS and TMB in the esophagogastric and colorectal cancer subgroups of MSK-GI cohort.

a-c In the esophagogastric cancer subgroup, Kaplan–Meier estimates of OS classified by GIPS (a) and the top 50% (b) and 20% (c) of TMB. **d-f** In the colorectal cancer subgroup, Kaplan–Meier estimates of OS classified by GIPS (d) and the top 50% (e) and 20% (f) of TMB.

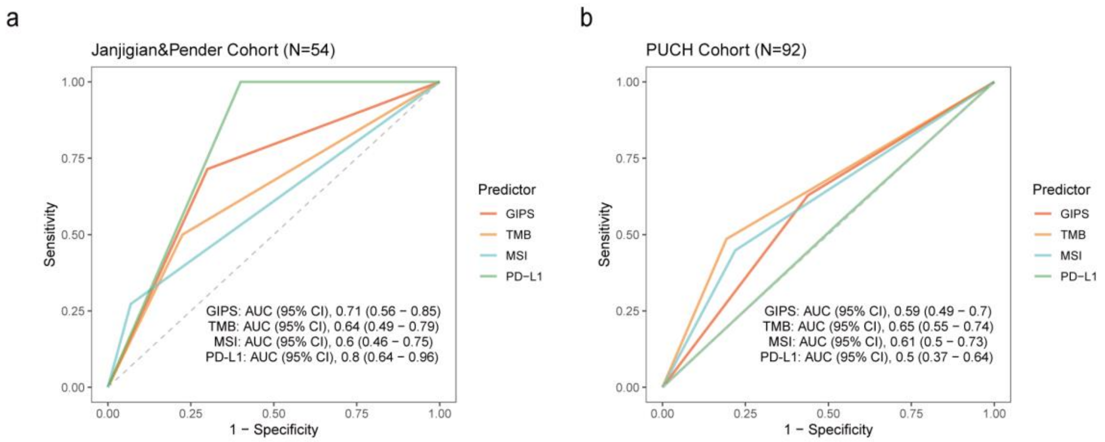

Supplementary Figure 3. ROC curves measuring the predictive value of the GIPS, TMB, MSI, and PD-L1 in the Janjigian & Pender (a) and PUCH cohorts (b).

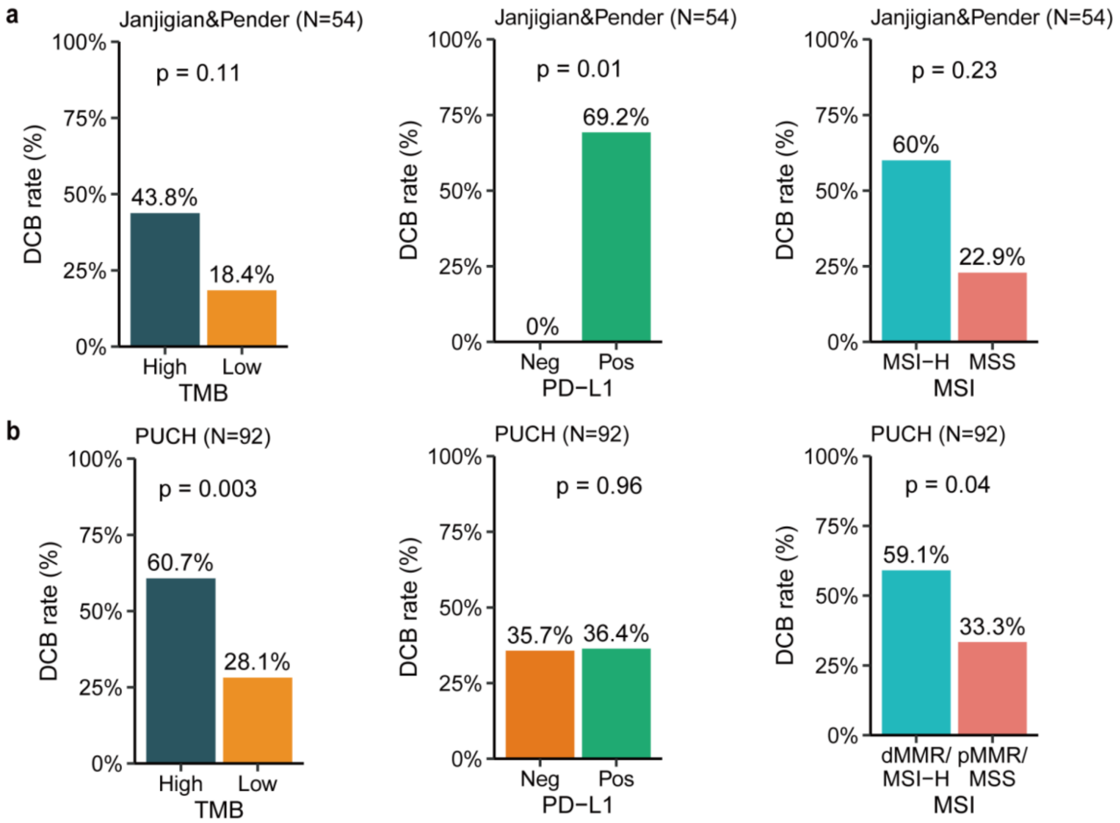

Supplementary Figure 4. Rate of durable clinical benefit in relation to potential biomarkers in two validation cohorts.

a and b In the Janjigian & Pender cohort (a) and PUCH cohort (b), the distribution of patients who achieved durable clinical benefit (DCB) and no durable benefit (NDB) according to TMB (left panel), PD-L1 (middle panel) and MSI status (right panel).

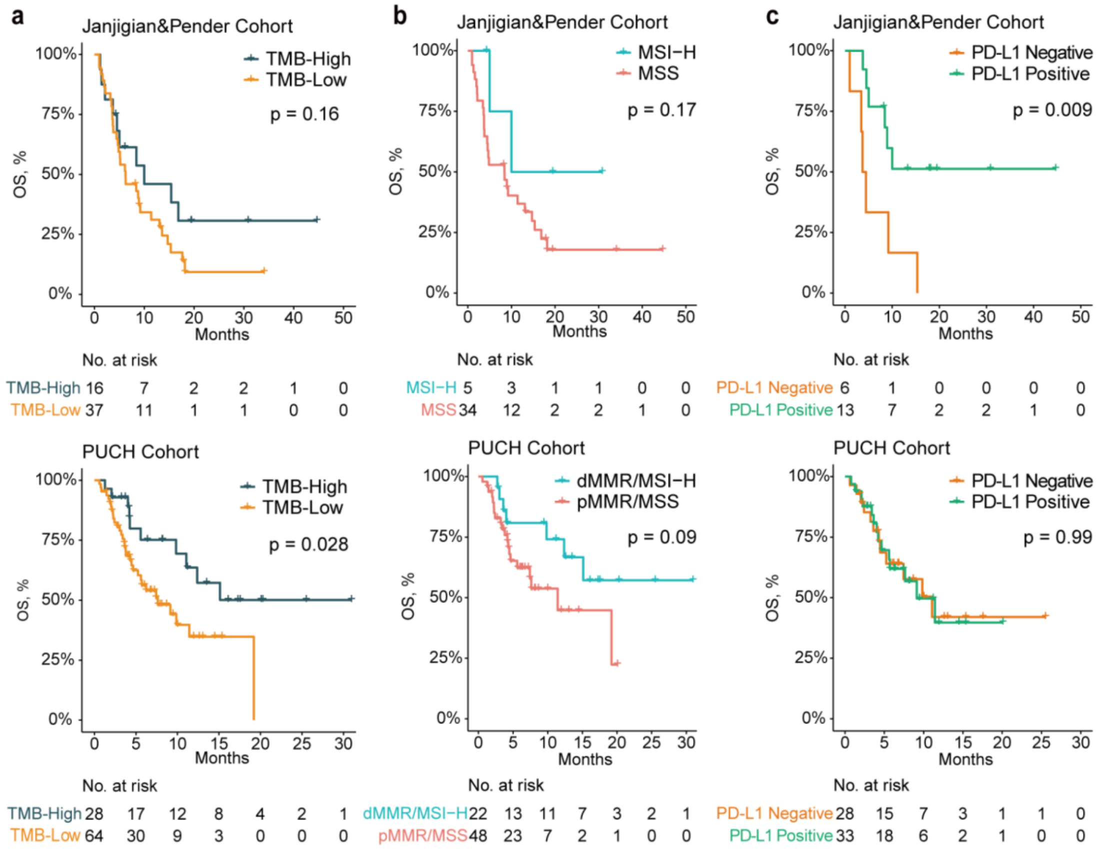

Supplementary Figure 5. Association of potential biomarkers and prognosis in the Janjigian & Pender cohort and PUCH cohort.

a-c Kaplan–Meier estimates of OS classified by TMB (a), MSI status (b) and PD-L1 expression (c) in the Janjigian & Pender cohort and PUCH cohort.

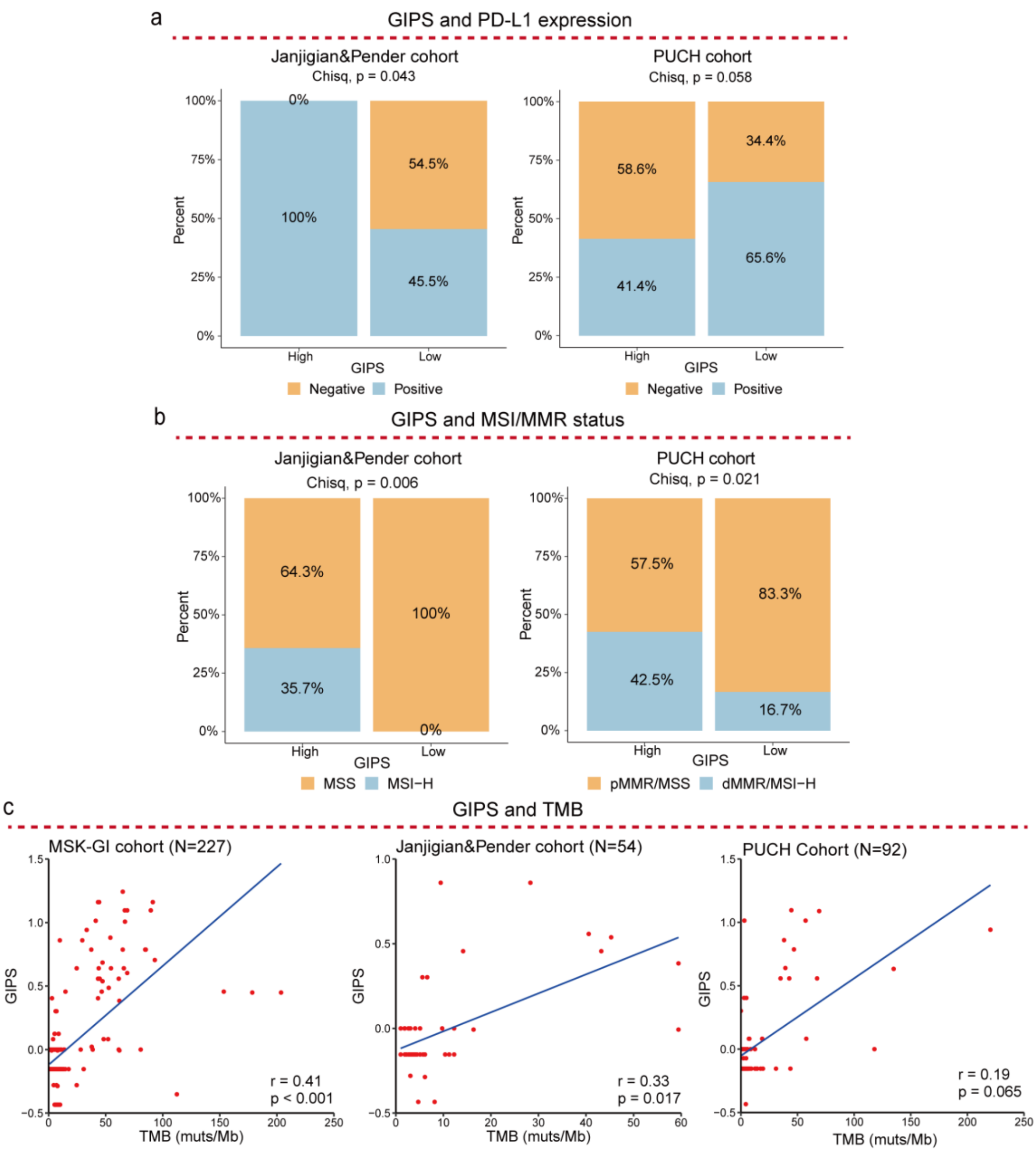

Supplementary Figure 6. Association between GIPS and each molecular factor in the three cohorts.

a The association between GIPS and PD-L1 expression. **b** The association between GIPS and MSI status. **c** The association between GIPS and TMB level.

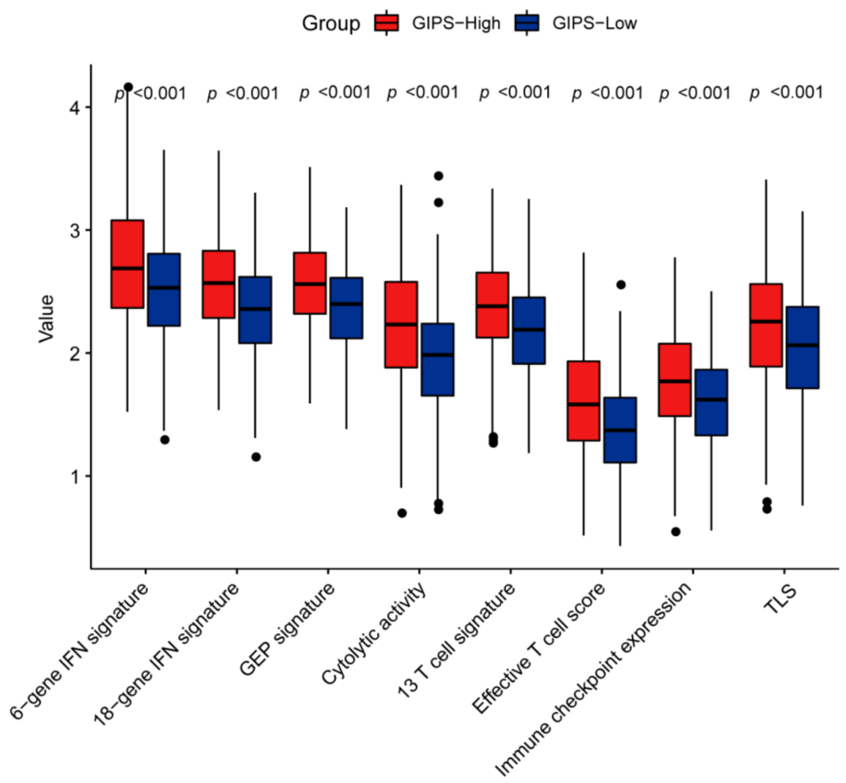

Supplementary Figure 7. Box plot of the immune-related signatures in comparison of the GIPS-high and GIPS-low groups of gastrointestinal cancers.
